# Envelope Protein-Targeting Zika Virus Entry Inhibitors

**DOI:** 10.3390/ijms25179424

**Published:** 2024-08-30

**Authors:** Abhijeet Roy, Qian Liu, Yang Yang, Asim K. Debnath, Lanying Du

**Affiliations:** 1 Institute for Biomedical Sciences, Georgia State University, Atlanta, GA 30303, USA; 2 Roy J. Carver Department of Biochemistry, Biophysics and Molecular Biology, Iowa State University, Ames, IA 50011, USA; 3 Lindsey F. Kimball Research Institute, New York Blood Center, New York, NY 10065, USA

**Keywords:** flavivirus, Zika virus, structural proteins, envelop protein, entry inhibitors

## Abstract

Zika virus (ZIKV; family, *Flaviviridae*), which causes congenital Zika syndrome, Guillain-Barré Syndrome, and other severe diseases, is transmitted mainly by mosquitoes; however, the virus can be transmitted through other routes. Among the three structural and seven nonstructural proteins, the surface envelope (E) protein of ZIKV plays a critical role in viral entry and pathogenesis, making it a key target for the development of effective entry inhibitors. This review article describes the life cycle, genome, and encoded proteins of ZIKV, illustrates the structure and function of the ZIKV E protein, summarizes E protein-targeting entry inhibitors (with a focus on those based on natural products and small molecules), and highlights challenges that may potentially hinder the development of effective inhibitors of ZIKV infection. Overall, the article will provide useful guidance for further development of safe and potent ZIKV entry inhibitors targeting the viral E protein.

## 1. Introduction

Zika virus (ZIKV) was first discovered in Uganda, Africa, in 1947 [1]. Initially, the virus was not considered to be a significant threat due to the limited number of reported cases and the mild symptoms it caused. Indeed, it had a limited impact on public health until the first outbreak occurred in the Yap Islands in the Pacific in 2007 [2]. ZIKV then emerged in French Polynesia in 2013, and subsequently spread to other Pacific Islands and the Americas, particularly Northeastern Brazil, where it caused a massive outbreak during 2015–2016 [3,4]. In February 2016, the World Health Organization (WHO) declared ZIKV a global public health emergency and emphasized the need for urgent measures to reduce transmission [5]. 

ZIKV is transmitted primarily by Aedes mosquitoes (Aedes aegypti and Aedes albopictus) [6]; however, it can also be transmitted through sexual contact, blood transfusions, and various body fluids, such as semen, breast milk, urine, saliva, and stool. There are even reported cases of laboratory-acquired transmission [7,8,9,10,11,12]. Pregnant women who are infected with ZIKV can transmit the virus to their developing fetuses [13]; thus, infection of pregnant women can have severe consequences, including spontaneous abortion, intrauterine growth restriction, microcephaly, leading to congenital Zika syndrome (CZS), and death of newborns is not uncommon [14,15]. CZS can manifest even in asymptomatic ZIKV-infected pregnant women [14,16,17]. ZIKV has a particular affinity for neural progenitor cells [18,19]; therefore, the virus exhibits neurotropic characteristics, resulting in severe neurological complications such as Guillain-Barré Syndrome (GBS) and meningoencephalitis [20]. GBS can manifest concurrently with or following ZIKV infection, and is usually sporadic, with most patients recovering completely [20,21]. 

Although transmission of ZIKV has declined since 2017, the virus still persists and could lead to significant outbreaks in some areas [8]. Around 1.62 million people in over 70 countries worldwide have been infected with ZIKV [22,23]. Currently, is circulating in approximately 89 countries, in which there is evidence of ongoing transmission via mosquitoes [24]. ZIKV is listed as a potential future pandemic virus by the WHO [25]. The discovery of congenital malformations such as microcephaly and GBS has gained significant attention and highlights the urgent need for continuous development of effective therapeutics [26]. 

## 2. ZIKV Replication and Life Cycle

Flaviviruses, single positive-stranded RNA viruses transmitted by arthropods, such as mosquitoes and ticks, belong to the *Flaviviridae* family, which also includes Dengue virus (DENV), West Nile virus, Yellow Fever virus, Japanese encephalitis virus (JEV), and Tick-borne encephalitis virus [27].

ZIKV replicates in the epithelial cells lining the mosquito’s midgut, eventually reaching the salivary glands. After an incubation period of about 10 days, the mosquito is capable of transmitting the virus through its saliva [28]. The virus enters the human body and infects dendritic cells before spreading to other organs via blood circulation. In humans, the incubation period for ZIKV infection is typically 3–12 days, and symptoms usually appear after 6–11 days [29]. In 99% of patients, ZIKV is cleared from the body within 24 days [30]. Because the virus replicates in the fetal brain for several months, pregnant women may experience prolonged viral infection, with symptoms intensifying during the early months of pregnancy [31].

**Figure 1:**
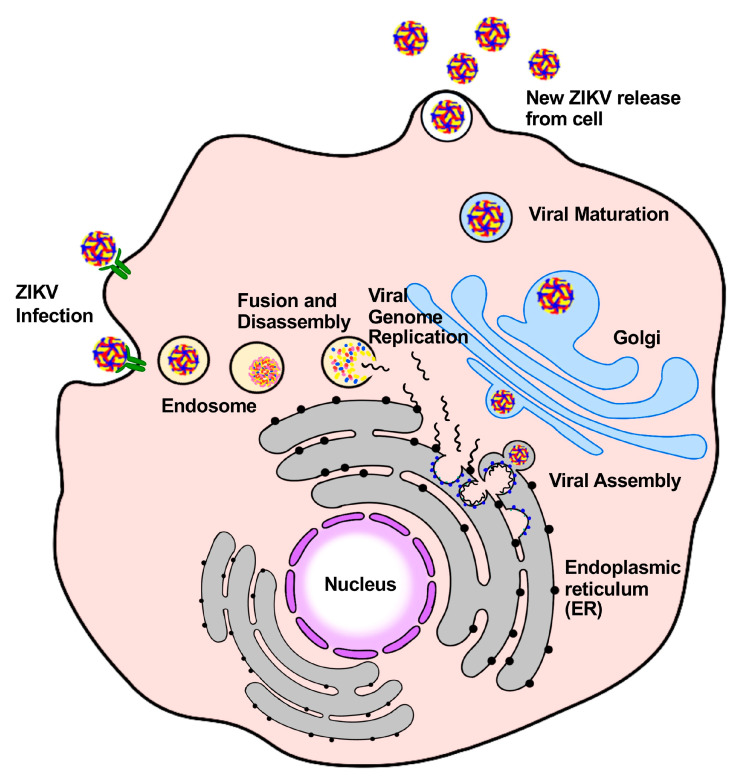
Schematic map of the life cycle of Zika virus (ZIKV). ZIKV binds host cells with several receptors and is internalized via clathrin-mediated endocytosis. The low pH in the late endosome status triggers the conformation change of the ZIKV E protein, resulting in the fusion between the viral and endosome membrane. The viral RNA is released into the cytoplasm, which is followed by translation and replication. Virus particles are assembled and bud from the endoplasmic reticulum (ER), and then maturate through the Golgi. Finally, mature viral particles are released from the cell.

Similar to other flaviviruses, ZIKV infection of host cells occurs via several key steps: receptor binding, entry, translation, replication, assembly, and release (Figure 1) [32]. Briefly, the envelope (E) protein aids viral attachment to host cell membrane receptors, followed by clathrin-mediated endocytosis, which allows the virus to be internalized. The viral membrane is then fused with the endosome membrane, thereby releasing the positive-strand viral RNA from the nucleocapsid into the cytoplasm [33]. Using the viral positive-strand genome as a template, viral RNA-dependent RNA polymerase (RdRp) synthesizes negative-strand genomic RNA. New viral positive-strand genomes and viral mRNAs are then transcribed on the surface of the endoplasmic reticulum (ER) using the negative-strand RNA as the template. Viral mRNA is then translated into polyproteins by the host cell machinery [34]. Both host and viral proteases cleave the newly translated polyprotein into functional proteins [35]. The newly synthesized positive RNA then integrates into the virions to start virus assembly in the ER. Immature virions mature in the Golgi apparatus through budding and are released into the extracellular environment [34].

## 3. ZIKV Genome and Proteins

**Figure 2:**
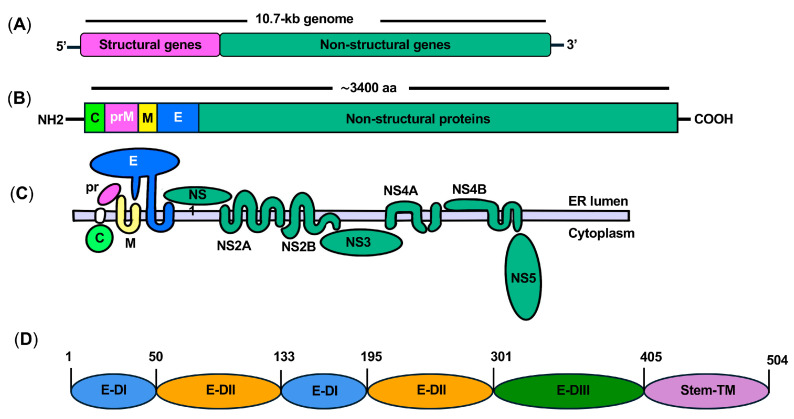
The genome and proteins of Zika virus (ZIKV). The genome of ZIKV is about 10.7 kilobase (kb), which is translated into a polyprotein of about 3400 amino acids (**A**,**B**). The polyprotein is cleaved to yield 3 structural (C, prM, E) and 7 non-structural proteins (NS1, NS2A, NS2B, NS3, NS4A, NS4B, and NS5) (**C**). Domain organization of E protein. E protein Domain I (E-DI); E protein Domain II (E-DII); E protein Domain III (E-DIII); stem and transmembrane regions (Stem-TM) of E protein (**D**).

Mature and immature ZIKV particles have a diameter of approximately 50 and 60 nm, respectively [36]. The positive-sense, single-stranded RNA genome is approximately 10.7 kilobases (kb) long and is flanked at the 5′ and 3′ ends by two untranslated regions [37]. Like other flaviviruses, the ZIKV genome lacks a 3′ poly(A) tail. A single open reading frame codes a polyprotein of about 3400 amino acids, which is processed by both viral and host proteases to yield three structural proteins and seven nonstructural proteins (Figure 2A) [35].

The three structural proteins, namely capsid protein (C), the precursor of the membrane (prM), and E protein, are the skeletal elements that form ZIKV particles (Figure 2B). The C protein is involved in virus assembly and release from infected cells; it also plays a key role by combining with the viral genomic RNA to create a nucleocapsid core that protects the viral RNA and maintains the structural integrity of the virus [38]. The membrane protein (M) is expressed as a glycosylated form (prM) and is associated with the lipid envelope derived from the host cell. The prM protein facilitates the proper folding and processing of the E protein. The prM and E proteins are viral surface glycoproteins that interact with the host cytoplasmic membrane. The E protein is responsible for the attachment of ZIKV to host cells, as well as the fusion of the viral membrane with the host cell membrane [39]. Generally, structural proteins play a role in host immunity by triggering immune responses and eliciting the production of antiviral antibodies. Understanding the functions of these structural proteins is important for developing strategies to target them and disrupt the life cycle of ZIKV [40].

The seven nonstructural proteins of ZIKV are NS1, NS2A, NS2B, NS3, NS4A, NS4B, and NS5 (Figure 2C); all have diverse functions in the ZIKV life cycle. They are involved in replication, assembly of the viral replication complex, evasion of the host immune response, and modulation of host cell functions [41]. For example, NS1 plays a crucial role in various aspects of ZIKV infection, including genome replication, pathogenesis, and modulation of the host immune response [42]. Within the host cell, NS1 collaborates with other nonstructural proteins to form a replication complex. Interactions between NS1 and these other viral proteins are crucial for the replication and pathogenicity of ZIKV [39]. NS2A has diverse functions, including facilitating viral RNA synthesis and virion assembly [43], and regulating innate immune evasion by inhibiting induction of type-I interferon [44]. NS3 is a vital component of the replication complex. The N-terminal region of NS3, in conjunction with its cofactor NS2B, forms a protease that is involved in cleaving the ZIKV polyprotein. This protease activity is essential for processing viral proteins during replication [39]; thus, the NS2B-NS3 protein complex is a prime target for drug screening. NS5 is another target for drug development [45]. It comprises an N-terminal RNA methyltransferase domain (MTase) and a C-terminal RdRp domain. The MTase domain is associated with the formation of the viral mRNA cap and facilitates translation, whereas the RdRp domain is responsible for RNA synthesis during genome replication [46]. 

## 4. Structure and Function of ZIKV E Protein

**Figure 3:**
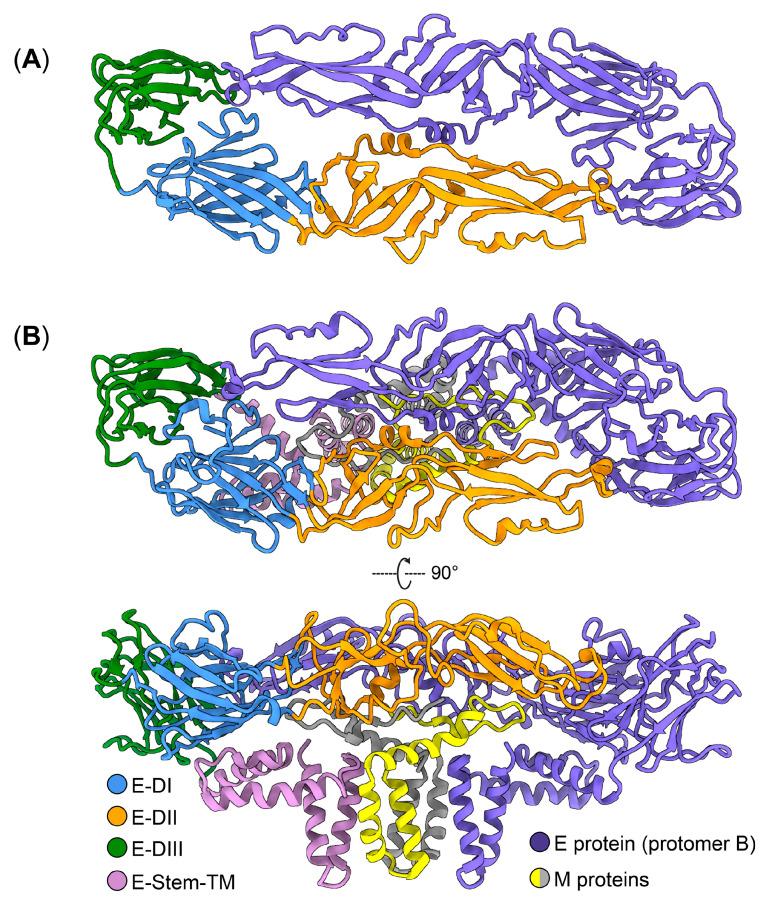
Structures of Zika virus (ZIKV) envelop (E) protein. (**A**) Crystal structure of ZIKV E protein dimer (PDB 5JHM). (**B**) Cryo-EM structure of ZIKV (E-M)_2_ protein complex (PDB 5IRE). E-DI, E protein Domain I; E-DII, E protein Domain II; E-DIII, E protein Domain III; E-Stem-TM, E protein stem–transmembrane domain.

Each ZIKV particle harbors 180 copies of the E protein, resulting in a compact virus particle with icosahedral symmetry. The E protein is a dimer consisting of two monomers with different domains (Figure 2D). Crystal and cryo-EM structures indicate that each copy of the E protein harbors three distinct domains within its ectodomain: Domain I (DI), Domain II (DII), and Domain III (DIII) (Figure 3A,B) [47,48]. DI contains the N-terminus of the E protein; it is a non-continuous β-shaped domain that serves as a link between DII and DIII. During ZIKV infection, DI is responsible for inducing essential conformational changes in the E protein [47]. DII is an extended finger-like structure containing the dimerization domain and a pH-sensitive fusion loop for viral fusion. A number of residues within DII form hydrogen bonds and electrostatic interactions, all of which help to stabilize the E-dimer protein [39]. DIII is an immunoglobulin-like domain located at the C-terminal end of the E protein and is responsible for attachment to target cells [49]. The DI, DII, and DIII domains of the E protein are connected to the viral membrane by two helices, known as stem anchors. These helices anchor the E protein to the viral membrane, thereby stabilizing the overall structure. DI is positioned at the center and is flanked by DII and DIII to form a monomer. The E monomers interact with adjacent monomers in an antiparallel fashion to form a dimer (Figure 3). The E protein undergoes conformational changes in response to variations in pH levels, particularly differences between the extracellular environment and endosomal environment within the host cell. These pH-induced changes are critical for the fusion of the viral and host cell membranes during the entry process [47]. The endosomal low-pH environment initiates a series of changes within the E protein, resulting in viral proteins entering a fusion-active state. ZIKV is found to be sensitive to endosomal pH levels of 6.1 to 6.5 for the fusion of E protein and endosomal membrane [50].

ZIKV E protein is essential for viral binding and entry. Entry of ZIKV into host cells typically begins with the binding of the virus to attachment factors on the cell surface. The surface of a mature ZIKV particle is covered by E protein homodimers arranged in an icosahedral format. This structural arrangement on the surface of the virus particle is essential for interactions with host cell receptors [47]. Individual interactions between ZIKV and human receptors are generally weak; however, when the virus comes into contact with multiple receptors simultaneously, the avidity increases, resulting in stronger attachment [33]. Multiple host cell receptors, including AXL (a receptor tyrosine kinase), DC-SIGN (dendritic cell-specific intercellular adhesion molecule-3-grabbing non-integrin), TIM (T-cell immunoglobulin and mucin domain), Integrins, and HSPGs (heparan sulfate proteoglycans), have been identified to mediate ZIKV entry into different target tissues, such as skin, dendritic cells, monocytes, testis, brain, and placental cells [33,51]. Initially, the DIII of the ZIKV E protein interacts with host cell receptors. After virus binding, the E protein undergoes distinct rearrangements to expose the fusion loop in DII. The viral membrane then fuses with the endosome membrane, thereby releasing the viral RNA into the cytoplasm. The process of fusion between the viral and endosome membranes is crucial for the release of viral genetic material [33,52]. 

Due to its role as a critical viral binding and entry target, as well as the presence of multiple epitopes, the E protein plays a key role in the immune responses against ZIKV. For example, it induces the generation of neutralizing antibodies, which recognize and bind to the E protein to neutralize the virus and block its function, thereby preventing the virus from infecting host cells [53]. Therefore, understanding the structure and function of this protein is essential for developing effective therapeutics, as well as vaccines and immunization strategies, against ZIKV.

## 5. Inhibitors of ZIKV E Protein-Mediated Entry

Since host cell entry is the first step of ZIKV infection, inhibiting entry mediated by the surface E protein is a key strategy underlying antiviral treatments. Currently, there are no licensed antiviral agents that target ZIKV specifically [54]. The ZIKV entry inhibitors at the preclinical stages are neutralizing antibodies designed to neutralize virus infection or designed as antiviral drugs to block viral attachment and other steps essential for viral entry. Screening compounds from diverse sources such as chemical libraries or existing drug databases is a common strategy used to identify molecules with antiviral activity [55]. The remainder of this review will discuss antiviral drugs as ZIKV entry inhibitors.

### 5.1. E Protein-Targeting Natural Products as ZIKV Entry Inhibitors

Natural compounds have been explored to identify potential antiviral agents against ZIKV. Natural products derived from plants, green tea, marine organisms, or other sources may exhibit antiviral properties, albeit with different mechanisms of inhibition (Table 1).

**Figure 4:**
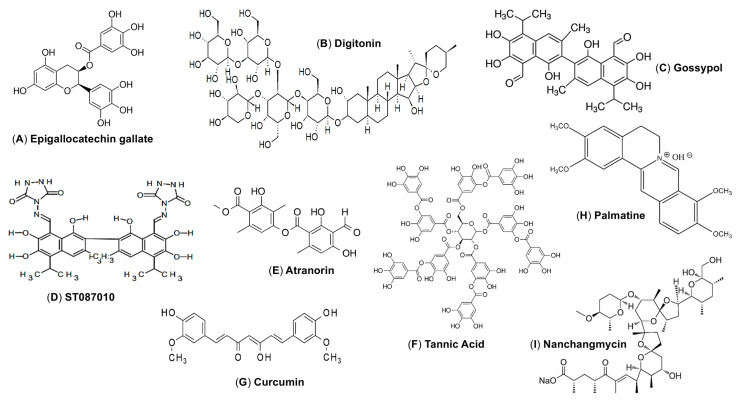
ZIKV E protein-targeting natural product compounds as viral entry inhibitors. (**A**–**I**) Chemical structures of the natural product compounds.

Most of the currently reported ZIKV entry inhibitors derived from natural products block viral entry by binding to the ZIKV E protein. For example, epigallocatechin gallate (EGCG) (Figure 4A), a type of polyphenol found in green tea, has antiviral activity against different strains of ZIKV. EGCG exerts its inhibitory effects binding to the ZIKV E protein, particularly the DI and DII regions, thereby preventing the conformational changes in DIII required for virus entry [56]. In addition to inhibiting ZIKV, EGCG also inhibits human immunodeficiency, herpes simplex virus, hepatitis C virus, and influenza virus [57,58,59]. Despite its antiviral properties, EGCG contains a catechol group that may lead to non-specific inhibition of various cellular targets. This lack of specificity could limit its efficacy as a targeted antiviral therapy [60]. Interestingly, EGCG can cross the placental barrier and be distributed throughout various fetal organs such as the brain and heart [61]. Digitonin (Figure 4B), an amphiphilic steroidal saponin belonging to the terpenoid class, exerts antiviral activity against various ZIKV strains by inhibiting the binding of the E protein to host cells [62]. Gossypol (Figure 4C), a polyphenolic compound with potent inhibitory effects against different ZIKV strains targets ZIKV directly by binding to the E protein, particularly the DIII region, with high affinity [62,63]. A gossypol derivative, ST087010 (Figure 4D), binds to the E protein with high affinity and blocks ZIKV entry in the same way as gossypol, but with more potency [63]. ST087010 also inhibits ZIKV infection in *Ifnar1*^−/−^ mice, preventing ZIKV-caused weight loss and fetal death, and increasing mouse survival, with significantly reduced viral titers in different organs, fetal brain, and amniotic fluid without showing toxicity [63]. Atranorin (Figure 4E), a natural product with antiviral activity against the hepatitis C virus [64], protects human glioblastoma cells from ZIKV infection, and targets the E protein directly, thereby disrupting virus entry and reducing infectivity [65]. Tannic acid (Figure 4F), which prevents ZIKV infection by binding directly to the E protein, induces a conformational change in several of the E protein residues essential for dimerization and membrane fusion [66].

**Table 1:** Summary of entry inhibitors targeting ZIKV E protein. CC_50_, 50% cytotoxic concentration; E, envelope; EC_50_, 50% effective concentration; IC_50_, 50% inhibitory concentration; IC_90_, 90% inhibitory concentration; ND, not determined; SI, selectivity index; ZIKV, Zika virus.

	E-binding Affinity (EC_50_)	Antiviral Activity (IC_50_) (ZIKV Strain)	Cytotoxicity (CC_50_)	Selectivity Index (SI)	Protection in Animals	Ref.
Epigallocatechin gallate (EGCG)	~119.94 kcal/mol	21.4 µM (ZIKV^BR^)	>200 µM	9	ND	[60]
Curcumin	ND	1.9 µM (HD78788)	11.6 µM	6	ND	[67]
Nanchangmycin	ND	0.1–0.4 µM (MR766 and Mex2-81)	~10 µM	ND	ND	[68]
Gossypol	7.12 µM	3.8 µM (PAN2016) 4.4 µM (R116265) 4.2 µM (PAN2015) 0.7 µM (FLR) 3.1 µM (R103451) 4.6 µM (PRVABC59)	14.5 μM	4.3	There is no protective efficacy in mice	[62,63]
ST087010	4.95 µM	3.2 µM (PAN2016) 2.9 µM (R116265) 3.5 µM (PAN2015) 2.8 µM (FLR) 3.6 µM (R103451) 2.4 µM (PRVABC59)	49.6 μM	17.1	Protects *Ifnar1*^−/−^ mice from infection of ZIKV (R103451 and PAN2026 strains)	[63]
Palmatine	ND	80 μM (IC_90_) (PRVABC59)	>100 µM	ND	ND	[69]
Digitonin	ND	4.31 µM (PAN2016) 6.52 µM (R116265) 5.0 µM (PAN2015) 3.34 µM (FLR) 4.3 µM (R103451)	56.29 µM	ND	ND	[62]
Atranorin	ND	10.39 µM (SZ01/2016)	>50 µM	>4	ND	[65]
F1065-0358	~8.314 kcal/mol (Docking score)	14 µM	>200 µM	>14	ND	[70]
ZINC23845959	9.35 Kcal/mol (Docking score)	3–5 µM (MR766)	>25 µM	ND	ND	[71]
ZINC23400466	4.77 Kcal/mol (Docking score)	3–5 µM (MR766)	>25 µM	ND	ND	[71]
ZINC12415353	6.15 Kcal/mol (Docking score)	3–5 µM (MR766)	>25 µM	ND	ND	[71]
3-110-22	2.1 μM	4.2 µM (IC_90_) (PF13/251013-18)	37.5 µM	~9	ND	[72,73]
3-110-2	ND	4.8 µM (IC_90_) (PF13/251013-18)	ND	ND	ND	
3-149-3	ND	14.9 µM (IC_90_) (PF13/251013-18)	ND	ND	ND	
LAS52154459	ND	5.13 µM (IC_90_) (PF13/251013-18)	98.11 µM	19	ND	[74]
Compound 16a	ND	2.61 µM (IC_90_) (INEVH116141)	23.83 µM	9.13	ND	[75]
Atovaquone	ND	2.1 μM IC_90_ (MR766)	ND	ND	ND	[76]

Other natural product-based ZIKV entry inhibitors inhibit the early stages of ZIKV infection, which also involve the E protein. For instance, curcumin (Figure 4G), a natural compound found in turmeric, shows its best inhibitory activity at the early stages of infection, suggesting that it blocks the binding of ZIKV to cell-surface receptors by interacting with the E protein. Curcumin inhibits ZIKV infection of different human cell lines, including HeLa, BHK-12, and Vero 6 cells, in a dose-dependent manner; however, the precise mechanism by which it prevents ZIKV binding to the cell surface remains unclear and needs further exploration [67]. Curcumin has poor bioavailability which limits its therapeutic potential, although several derivatives do show enhanced bioavailability and inhibitory activity [77]. A protoberberine alkaloid compound, palmatine (Figure 4H), also inhibits ZIKV infection during the early stages. In particular, it affects binding of ZIKV binding to host receptors, as well as virus entry and stability, by interacting with the E protein. Molecular docking analysis indicates that palmatine binds to the ZIKV E protein with low background cytotoxicity [69].

Natural products inhibit ZIKV entry via many alternative mechanisms. Nanchangmycin (Figure 4I), a bacterial-derived polyether antibiotic prevents ZIKV from entering and infecting cells by blocking clathrin-mediated endocytosis by several human cell types, including human U2OS cells, Jeg-3 cells, and human brain microvascular endothelial cells. In addition to ZIKV, Nanchangmycin also shows antiviral activity against Chikungunya and Sindbis viruses, both of which use the same internalization pathway as ZIKV [68]. 

### 5.2. E Protein-Targeting Small Molecules as ZIKV Entry Inhibitors

Similar to natural product-derived entry inhibitors, ZIKV entry inhibitors are based on small molecules that bind to the E protein. They operate by blocking conformational changes in the E protein or by inhibiting membrane fusion mediated by the E protein (Table 1).

**Figure 5:**
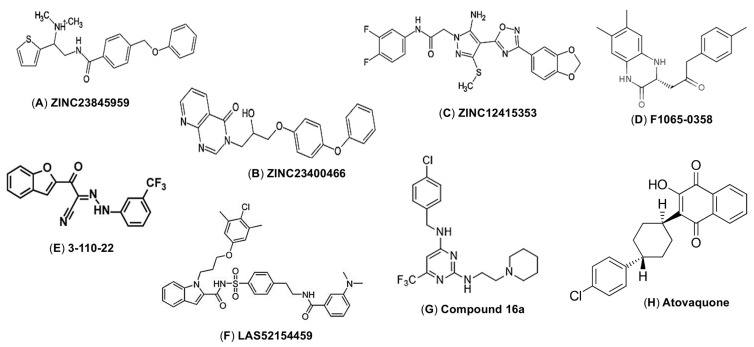
ZIKV E protein-targeting small molecules as viral entry inhibitors. (**A**–**H**) Chemical structures of the small molecules.

ZINC23845959, ZINC23400466, and ZINC12415353 (Figure 5A–C), which were identified by homology modeling and similarity-based screening, bind to the E protein via a β-octyl glucoside binding site and demonstrate significant inhibition of ZIKV-infected Vero cells. The molecular docking analysis predicted that the compounds interact with the residues at position Thr48, Thr49, Val50, Tyr99, His206, Glu272, Arg279, Phe281, and Ser282 of ZIKV E protein, thus directly preventing the E-mediated entry [71]. A reporter ZIKV particle-based infection assay identified synthetic carbohydrate receptors (SCRs), which are non-biological derived small molecules, as potential ZIKV entry inhibitors; such compounds show a 50% inhibitory concentration (IC_50_) as low as 0.16 μM, with minimal toxicity at high concentrations [78]. These SCRs inhibit the early stages of ZIKV infection, likely by inhibiting binding between the ZIKV and cell-surface glycans, thereby preventing viral entry [78]. 

A structure-based virtual screening approach revealed that the small molecule compound F1065-0358 (Figure 5D) effectively inhibits ZIKV replication in Vero cells. All-atom molecular dynamic simulations confirmed its stable binding to the E protein, which prevents trimerization of the E protein during the fusion process. F1065-0358 interacts with highly conserved residues within the DI domain at residues His144 and Phe183, and in DIII at Lys301, Tyr305, and Asn362, thereby interfering with its conformational rearrangement. This small molecule has the potential to inhibit other flaviviruses [70]. Cyanohydrazone series 3-110-22 (Figure 5E), 3-110-2, and 3-149-3 target a pocket located at the interface between DI and DII of the ZIKV E protein (which is essential for the conformational change in the E protein at low pH) block entry of ZIKV and other flaviviruses, such as DENV and JEV, albeit with relatively low antiviral activity [72]; however, compound 3-110-22 binds to ZIKV E protein with high affinity [73]. 

After using a competitive amplified luminescent proximity homogeneous assay to screen two libraries comprising nearly 27,000 compounds, LAS52154459 (Figure 5F) was identified; however, its antiviral activity is relatively low. The compound works by inhibiting E protein-mediated membrane fusion [74]. A pyrimidine derivative named compound 16a (Figure 5G), which was identified using a structure-based computer-aided drug design, prevents ZIKV infection, again with low antiviral activity (IC_50_). It inhibits ZIKV infection at the early stages, especially the virus internalization and post-internalization steps, suggesting that it inhibits the E protein-mediated membrane fusion step. This compound has a favorable bioavailability and safety profile, and it also inhibits DENV infection [75]. Screening of an FDA-approved drug library using a quantitative mosquito-cell-based membrane-fusion assay identified Atovaquone (Figure 5H) as a ZIKV inhibitor that blocks ZIKV infection, as well as that of four DENV distinct serotypes, suggesting its potential as a pan-flavivirus entry inhibitor. This small molecule targets membrane fusion induced by the E protein of various flaviviruses [76]. 

## 6. Host Factors That Modify the ZIKV E Protein

Several host factors have the potential to target the ZIKV E protein and block viral infection. Ubiquitin-specific peptidase 38 (USP38), a member of the ubiquitin-specific processing enzyme family, is identified as a histone deubiquitinase. USP38 has been found to inhibit type-I IFN signaling during viral infection [79], playing an important role in host resistance to ZIKV infection [80]. The post-translational modifications of the E protein have a significant impact on ZIKV replication, infection, and transmission [36]. It has been evident that HeLa cells overexpressing USP38 significantly reduced the ubiquitination of E protein, thus inhibiting ZIKV infection. Additionally, mutant cells lacking the deubiquitinating activity of USP38 enhanced ZIKV invasion and infection. A co-immunoprecipitation experiment showed that USP38 bound to the ZIKV E protein, specifically with DI, DII, and C-terminal domain, and attenuated its K48-linked and K63-linked polyubiquitination, thereby repressing infection [80]. Palmitoylation is a post-translational modification of proteins that is mediated by a series of enzymes named ZDHHC1–ZDHHC9, and ZDHHC11–ZDHHC24 [81]. Among these, ZDHHC11, which interacts with the ZIKV E protein and catalyzes its palmitoylation, is crucial for E protein stability, trafficking, and functionality. A study confirmed that a palmitoylation inhibitor 2-bromopalmitate enhanced ZIKV infection, which subsequently led to the identification of ZDHHC11 as a key enzyme of E protein palmitoylation [82]. Overexpression and knock-out studies revealed that overexpression of ZDHHC11 inhibited ZIKV infection in HEK293T cells, whereas knock-out enhanced the ZIKV infectivity. These results suggest that ZDHHC11 has anti-viral activity against ZIKV, possibly through palmitoylation of E protein at the Cys308 position [82]. These host factors could serve as host protein therapeutic targets that prevent and treat ZIKV infection.

## 7. Potential Challenges of ZIKV E-Targeting Entry Inhibitors

ZIKV is a significant public health concern due to its potential to cause severe neurological complications such as microcephaly in newborns. The E protein plays a vital role in viral entry and fusion, making it an appealing target for antiviral treatments; however, inhibiting the E protein comes with various challenges and limitations. 

ZIKV virions are released from host cells as a mixture of immature, partially mature, and mature particles [83]. These different conformations impact the efficacy of therapeutics targeting the E protein. Moreover, the mature E protein is always in dynamic motion, known as “virus breathing”, which is temperature-dependent and strain-specific [84]. These two factors present a great challenge for accessing epitopes on the E protein. The E protein of ZIKV also undergoes N-linked glycosylation, which can alter its structure and function. Different forms of glycosylation impact its interactions with other molecules and potentially affect viral pathogenesis. This diversity in glycosylation patterns adds a layer of complexity to the task of targeting the E protein for therapeutic purposes [85,86,87]. 

During viral entry, the E protein interacts with the M protein, which is a crucial step in the fusion process. Disrupting this interaction could halt viral fusion and replication. Nevertheless, targeting this specific interaction poses challenges due to its intricate nature and the risk of unintended effects on other viral processes [88,89]. Some inhibitors may impact other cellular processes, thereby limiting their therapeutic potential. Certain inhibitors could affect the virus through various mechanisms such as viral replication, assembly, or egress, rather than by specifically targeting the E protein [72]. This lack of specificity may complicate the development of targeted therapeutics that specifically inhibit the E protein. In addition, outbreaks of flaviviruses often overlap; in particular, ZIKV and DENV co-infection have been observed in areas in which these two viruses are endemic [90,91]. Cross-reactivity can result in unintended effects, with potential treatment resistance posing a challenge for developing therapeutics targeting the E protein. 

Overall, the high degree of E protein conservation, the possibility of off-target effects by inhibitors, and the complex mechanisms involved contribute to the difficulty in developing effective and specific inhibitors of ZIKV entry. Overcoming these challenges will require further research to identify targeted therapeutics capable of effectively inhibiting ZIKV infection. 

Computational approaches, such as molecular simulation and in silico design, are effective tools for exploring the interactions between viral proteins and potential drug candidates at an atomic level. The use of electrostatic forces to study virus and drug interactions is a fundamental aspect of molecular simulations and drug design [92,93]. Of note, understanding of the dynamical behavior and flexibility of ZIKV E protein can aid the design of drugs against ZIKV. Potential drug-binding sites on the ZIKV E protein have been identified using molecular dynamics simulation studies [94]. Characterization of different epitopes on the DIII region of ZIKV E protein using this approach reveals that epitopes binding to moderately or weakly neutralizing antibodies are highly flexible, whereas those binding to highly neutralizing antibodies are rigid [95]. Therefore, computational methods would be pivotal in optimizing the design of entry inhibitors targeting the E protein of ZIKV and overcoming the potential challenges to some extent.

## 8. An Overview of ZIKV Vaccines Based on the E Protein

Some E protein-based ZIKV vaccines, including DNA, mRNA, and viral vectored vaccines, have shown promising results in preclinical studies, several of which have been moved into human clinical trials [96,97]. DNA vaccines containing ZIKV prM and E genes for encoding prM and E proteins have shown effective protective efficacy against ZIKV. A Phase II clinical trial (NCT03110770) has been completed for a DNA vaccine (VRC5283), which encodes prM-E proteins of ZIKV. Phase I clinical trials have been completed for several mRNA vaccines, including mRNA-1325 and mRNA-1893 encoding ZIKV prM-E proteins [98,99]. A variety of viral vectored vaccine platforms have been developed, several of which have been processed into clinical trials [99]. For example, a Chimpanzee adenovirus-based vaccine, ChAdOx1 ZIKV, which incorporates prM-E protein proteins of ZIKV, generates neutralizing antibodies, resulting in protective efficacy in animal models [100]. A Phase I clinical trial of ChAdOx1 ZIKV has been completed to evaluate its safety and immunogenicity (NCT04015648).

## 9. Conclusions and Future Perspectives

This review describes the life cycle, genome structure, and proteins of ZIKV, focusing on the structure and function of the E protein. We also provide an overview of E-targeting entry inhibitors and describe their mechanism of inhibition. Of note, most of the currently developed ZIKV entry inhibitors show only in vitro inhibitory activity. The in vivo protective efficacy of these inhibitors still needs to be elucidated, and their inhibitory efficiency needs to be improved. In particular, combined treatment with inhibitors that act via different mechanisms, or target different binding sites on the ZIKV E protein, will likely increase the overall inhibitory activity of entry inhibitors. Taken together, the data suggests that safe and effective inhibitors need to be developed to prevent and treat future ZIKV pandemics or outbreaks. 

## Data Availability

Not applicable.
